# Malaria is associated with poor school performance in an endemic area of the Brazilian Amazon

**DOI:** 10.1186/1475-2875-8-230

**Published:** 2009-10-16

**Authors:** Sheila Vitor-Silva, Roberto C Reyes-Lecca, Tamam RA Pinheiro, Marcus VG Lacerda

**Affiliations:** 1 Escola Superior de Ciências da Saúde, Universidade do Estado do Amazonas, Av Carvalho Leal, 1777, 69065-001, Manaus, Brazil; 2 Núcleo de Medicina Tropical, Universidade de Brasília, CP 4356, 70919-970, Brasília, Brazil; 3 Centro Universitário Nilton Lins, Av Professor Nilton Lins, 3259, 69058-040, Manaus, Brazil; 4 Gerência de Malária, Fundação de Medicina Tropical do Amazonas, Av Pedro Teixeira, 25, 69040-000, Manaus, Brazil

## Abstract

**Background:**

Approximately 40% of the world's population is at risk for malaria. In highly endemic tropical areas, malaria is a major cause of morbidity and mortality during infancy. There is a complex interrelationship between malaria, malnutrition and intestinal helminths, and this may impair cognitive development in children. The aim of this study was to determine the relationship between malaria and school performance in children living in an endemic area where *Plasmodium vivax *is the species responsible for most of the cases.

**Methods:**

The study was conducted in the Municipality of Careiro, Amazonas, Brazil, with five to14 year-old children, studying the first eight grades of public school, during the year 2008. After an initial active case detection, during nine months of follow-up, passive malaria cases detection was instituted, through a thick blood smear performed in every child with fever. School performance was evaluated by the final notes in Mathematics and Portuguese Language. Performance was considered poor when either of the final notes in these disciplines was below the 50^th ^percentile for the respective class and grade.

**Results:**

The total number of students followed-up in the cohort was 198. Malarial attacks were reported in 70 (35.4%) of these students, with no cases of severe disease. *Plasmodium vivax *was detected in 69.2% of the attacks, *Plasmodium falciparum *in 25.5% and both species in 5.3%. In the multivariate analysis, adjusting for age, mother's education, time living in the study area and school absenteeism, presenting with at least one episode of malaria independently predicted a poor performance at school [OR = 1.91 (1.04-3.54); p = 0.039].

**Conclusion:**

Non-severe malaria compromises the school performance of children even during a nine-month follow-up, potentially contributing to the maintenance of underdevelopment in countries endemic for malaria. This is the first evidence of such impact in Latin America, where *P. vivax *is responsible for the majority of the cases.

## Background

Approximately 40% of world's population live in areas that have some risk of malaria infection. Every year more than 500 million people are infected, particularly children, and they are more susceptible to severe manifestations of the disease [1]. The majority of cases and deaths occur in sub-Saharan Africa, but Southeast Asia, Latin America and Eastern Mediterranean areas are also affected [2]. Brazil reports most of the morbidity attributed to malaria in the Americas, with the disease being responsible for a substantial decrease in the quality of life in this hemisphere.

Despite the implementation of malaria control strategies in Brazil, children are still affected by the disease on a large scale. In 2008, the annual parasite index (API) in older than 14 years-old was 11 cases/1,000, however the API for children from five to 14 years of age was 14.3/1,000 [4]. Most of children living in endemic areas suffer multiple episodes of malaria before they become adults. In general, these episodes are acute, not complicated, and after adequate treatment the children's recovery is apparently satisfactory, especially in areas where *Plasmodium vivax *is frequent [5].

Studies in children with severe falciparum malaria (mostly cerebral malaria) focusing on neurological sequelae show some impairment in developing cognitive abilities after the acute episode, both in the short-term [6-8] and the in long-term [9,10]. Furthermore, as suggested by Kihara *et al *[11], deficits in all categories of cognition (attention, memory, visual-spatial skills, language and executive functions) may occur after severe *P. falciparum *infection, but also appear to occur after less severe infections. The effects of cumulative or repetitive episodes on cognitive development of children need more clarification, as well as information about the effects of *P. vivax *infection on cognition, the major parasite found in the Americas.

Considering that Brazil has more *P. vivax *(84.4% of the reported cases in 2008) than *P. falciparum *infections, this cohort study was conducted in an unstable transmission endemic area for malaria in the Western Brazilian Amazon, where severe malaria is rarely seen. The principal aim was to assess whether the occurrence of malarial episodes independently influences a child's performance in school.

## Methods

### Study area and population

**Figure 1:**
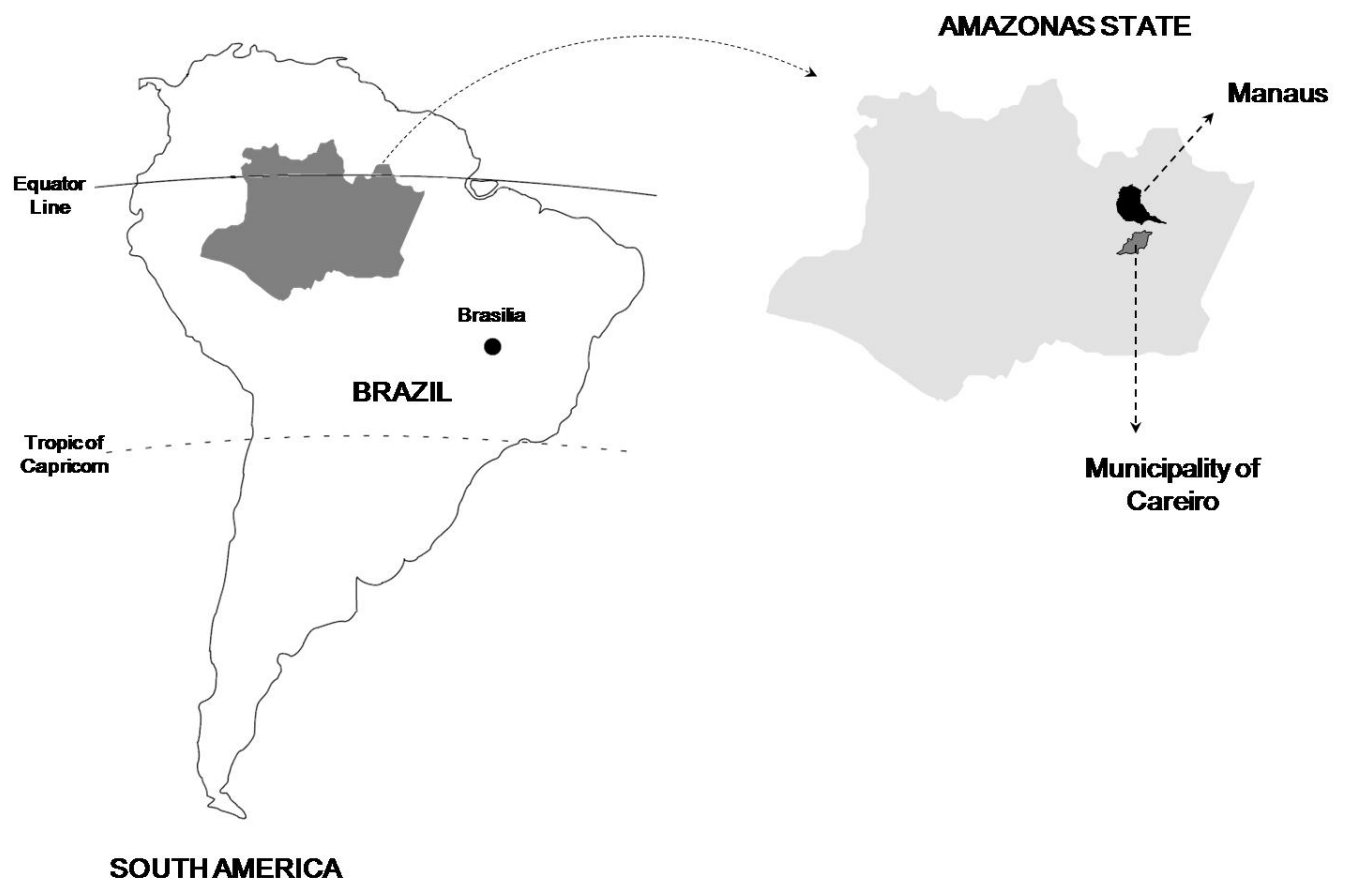
**Geographic localization of the Municipality of Careiro, Amazonas, Brazil**.

The Municipality of Careiro, in the Amazonas State (Western Brazilian Amazon), 112 km from the capital of the state, Manaus (Figure 1), reported 5,238 cases of malaria in 2008, with 1,127 (21.5%) cases in children five to 14 years-old of age. The local API during the same year was 180.9/1,000 inhabitants [12]. The research was conducted in two areas of recent occupation devoted to agriculture (Panelão and Céu Azul Communities), with a total population of 736 persons (census performed immediately before the beginning of the study), of which 208 were children between five to 14 years of age.

### Study design

Students from two schools in grades 1-8, between 6-14 years old were followed from March to December 2008 in a cohort. In March, active case detection of malaria through thick blood smear and the haemoglobin (Hb) concentration were performed in all children from the schools (initial cross-sectional evaluation). Children positive for *Plasmodium *were treated according to the Brazilian Anti-Malarial Treatment Guidelines [13]. In the case of *P. vivax *malaria, chloroquine is used for 3 days (10 mg/kg on day 1 and 7.5 mg/kg on days 2 and 3) followed by primaquine (0.5 mg/kg/day for 7 days). In the case of *P. falciparum *malaria, artemether/lumefantrine is prescribed for three days as the first-line regimen. During the follow-up period, children with symptoms of malaria submitted a thick blood smear for the diagnosis of malaria (passive case detection). All the teachers were informed about the objectives of the study and reported school absenteeism to the investigators.

### Laboratory diagnosis of malaria

Two thick blood smears were collected on the same slide, stained with Giemsa and examined under 1,000× magnification by the local microscopist in a laboratory of each community. The microscopists were formally and regularly trained according to the standard methods of slide reading. A minimum of 100 microscopic fields were examined for the final diagnosis of a negative smear.

### Haemoglobin concentration

Haemoglobin concentration was measured in venous blood obtained from digital puncture, using a portable HemoCue^® ^photometer (Anglholm, Sweden), in the cross-sectional evaluation.

### Assessment of school performance

Two disciplines were selected to evaluate the school performance: Portuguese Language and Mathematics, because both are taught to children in all of the studied grades. To assess school performance the teacher's evaluation of each classroom was used, which is based on the evaluating system recommended by the Brazilian Ministry of Education (the range of the final score is from 0 to 100). In each grade, a student was considered to have a poor performance if his final score in Portuguese Language and/or Mathematics was under the 50^th ^percentile for his classroom and grade. This was done to avoid the biases of evaluation by different teachers with distinct levels of qualification with the intent to compare children with their colleagues and year group.

### Nutritional status assessment

Weight and height were obtained by internationally recommended methods [14]. Weight was measured by use of a digital scale and height was assessed by a single observer with the help of a tape. Body Mass Index (BMI) was calculated using the program EPI-INFO 3.4.3. BMI Z-score < -2 was defined as malnutrition, scores between -2 and Z < - 1 as the risk zone; Z score between - 1 and 2 normal weight, and Z score > 2 as obesity.

### Data analysis

A database was created in SPSS 16.0 software (SPSS, Chicago, IL). Proportions were compared with chi-square test, using Fisher exact test when necessary. The crude *Odds Ratio *(OR) with its respective 95% Confidence Interval (95% CI) was determined considering the school performance as the dependent variable, with a statistical significance at the level of 5%. Logistic regression was used for the multivariate analysis and the adjusted *Odds Ratio *with 95% CI was also calculated.

A stepwise procedure for selecting variables in the multivariate analysis was used.

### Ethical considerations

The study protocol was approved by the Ethics Review Board of the Tropical Medicine Foundation of Amazonas (approval number 0656/2008). Both the Municipal Secretariat of Education and the school managers allowed the performance of the study. After educations regarding the objectives of the project, parents or guardians signed an informed consent.

## Results

A total of 198 students were enrolled, 105 (53.0%) male and 93 (47.0%) female. In the preliminary cross-sectional evaluation, nutritional status was examined in 170 children, resulting in 143 (84.1%) with normal status, 20 (11.8%) in risk of malnutrition and 7 (4.1%) with malnutrition. Only one asymptomatic child was positive for *P. falciparum *and was treated with oral artemether/lumefantrine. During the passive case detection, malarial attacks were registered in 70 (35.4%) students. 46 of these had only one malarial attack during the follow-up period, 21 children had two attacks and three children had three attacks. Considering all the malarial episodes, *P. vivax *was the involved species in 69.2%, *P. falciparum *in 25.5%, and both species in 5.3%. No severe case was clinically detected and no child needed hospitalization. At the end of the cohort follow-up, overall poor performance was detected in 94 (47.5%) school children.

Table 1 shows the odds ratios and 95% CI for bivariate and multivariate analysis. In the bivariate analysis no significant differences were seen in independent variables such as sex, age, nutritional status, time living in the study area, anaemia at enrolment and school absenteeism between children with and without poor performance. An exception was an increased proportion of more educated mothers and at least one malarial episode in the poor performance group. In the multivariate analysis, in a model adjusted for age, mother's education, time living in the study area and school absenteeism, the malarial attacks were independently associated with the odds of poor performance at school. During the follow-up interval, none of 12 teachers employed by the schools suffered a malarial attack.

**Table 1:** Bivariate and multivariate analysis of independent variables with the school performance in Portuguese language or mathematics in a cohort of 198 children followed from March to November 2008, in two schools from an endemic area for malaria (Careiro, Amazonas, Brazil).

		**Poor performance in language and/or mathematics**					
		**Yes n (%)**	**No n (%)**	**Crude *Odds Ratio *(95% CI)**	**p**	Adjusted *Odds Ratio *(95% CI)*	**p**
Sex	Male	49 (52.1)	56 (53.8)				-
	Female	45 (47.9)	48 (46.2)				
Age	6-10 years	54 (57.4)	48 (46.2)	0.64 (0.36-1.11)	0.112	1.70 (0.94-3.04)	0.077
	11-14 years	40 (42.6)	56 (53.8)				
At risk of or with malnutrition**		16 (17.0)	11 (14.5)	1.21 (0.53-2.79)	0.651	-	-
Mother's education	< 5 years	59 (62.8)	80 (76.9)	1.98 (1.02-3.85)	0.030	0.56 (0.30-1.06)	0.076
	≥ 5 years	35 (37.2)	24 (23.1)				
Time living in the study area	< 5 years	70 (74.5)	78 (75.0)	1.03 (0.54-1.95)	0.931	0.96 (0.49-1.90)	0.906
	≥ 5 years	24 (25.5)	26 (25.0)				
Anaemia^# ^at enrollment**		21 (25.0)	23 (24.0)	1.06 (0.54-2.09)	0.871	-	-
School absenteeism throughout the follow-up interval	Until 1 week	62 (66.0)	64 (61.5)	0.83 (0.46-1.48)	0.519	1.20 (0.65-2.20)	0.559
	> 1 week	32 (34.9)	40 (38.5)				
≥ 1 malarial attacks during the follow-up		41 (43.6)	29 (27.9)	2.00 (1.11-3.62)	0.021	1.91 (1.04-3.54)	0.039

Total		94 (100)	104 (100)	-	-	-	-

* Logistic regression model using age, mother's education, time living in the study area, school absenteeism and malarial attacks as independent variables

** n = 170

^# ^Hb < 11,0 g/dL

## Discussion

School performance in the native language (Portuguese) and/or Mathematics in a cohort of 198 children five to 14 years-old was associated with at least one episode of malaria during a nine-month follow-up interval, in an area highly endemic for *P. vivax *malaria.

Fernando et al [5], working with children at the same age living in malaria endemic areas in Sri Lanka, where *P. vivax *was also the major species, found the same association. Working with five to six year-old children, they also observed that the performance at school entry was poorer in children with history of five or more malarial attacks [15]. These results suggest that repeated malarial attacks are able to produce a negative and cumulative effect on school performance. In this study, the information on past episodes was not assessed due to imprecise and potentially invalid information given by the parents, according to information given in previous studies performed in this same study area. Due to the unstable transmission of the disease in this study area, in a nine-month follow-up period, it was not possible to evaluate the impact of cumulative malarial episodes on school performance.

Children's cognitive development can be influenced by many factors, such as morbidity by infectious diseases, nutritional status (notably iron deficiency), anaemia, parental education, school absenteeism and other social, economical and environmental factors [16-19]. In the present study, when potential confounders were controlled (age, mother's education, school absenteeism and time of residence in the area), malaria infection still was found to be an independent predictor of poor performance. In the study area, as in other agricultural settings in the Brazilian Amazon, families share homogeneous living conditions and income status, as well as the same public schools. This could explain the lack of influence of many of the variables. In non-endemic areas for malaria in Brazil, the nutritional and the socioeconomic status were associated with failure in school [20]. Anaemia is not infrequent in patients with malaria in the Brazilian Amazon [21], however, malaria-triggered anaemia as the cause of this poor performance needs further study with an appropriate design and a model in which potential contributing factors to anaemia are ruled out.

Low mother's education has been considered as a risk factor for severe malaria in African children [22], but for non-severe malaria, it did not influence school performance independently [5], similar to the present observations. It is possible that the mother's education was associated with poor school performance in the bivariate analysis due to the fact that these more educated mothers (most of them re-started to study recently in these communities' schools) are also more absent from their homes and therefore do not participate in their children's education.

School absenteeism is described as a relevant outcome of malarial infection, but it is still controversial. In Kenyan endemic areas, severe malaria was responsible for 3-8% of school absenteeism [23]. On the other hand, when all clinical forms are considered, malaria can be responsible for up to 36% of absenteeism [24]. In this study absenteeism was not more frequent among children with malaria, possibly because in the school students have free access to food and a better health provisions.

Effects of malaria infection on cognition can be divided into direct effects of cerebral injury, as a consequence of severe malaria, and indirect potential impacts on performance mediated by chronic infection, repetitive infections and associated anaemia/malnutrition [25-27]. No severe case of malaria was observed during the study, even in children infected by *P. falciparum*. This is not unexpected in the Brazilian Amazon, with predominant *P. vivax *infections and easy and free access to diagnosis and treatment. In this area, therefore, the poor performance in school reflects essentially the effects of non-severe malaria, which is increasing in Latin America [28].

In a similar endemic area in the Brazilian Amazon (Acre State), malaria morbidity was strongly associated with land clearing and farming [29]. The impact of malaria in this population is still largely unknown. An effort to understand the burden of this infection in these agricultural activities could substantially motivate policy development to mitigate the social impact of malaria.

There is scarce evidence that asymptomatic parasitaemia affects children's cognition in the long-term. When asymptomatic carriers of *Plasmodium *are treated, students improve their attention at school [30].

Malaria is still considered a poverty related disease, affecting primarily people with the lowest socioeconomic status and difficult access to health services, leading to long-term impairment of health and education [31,32]. Many African countries have 1-2% of their gross domestic product at risk because of working-age people with malaria [33]. It was demonstrated for the first time in Latin America that malaria may affect children's cognition, potentially maintaining poverty in such endemic areas. This is distinct from endemic areas in Africa, where malnutrition is more frequent and socioeconomic status is lower.

Strictly urban areas would be affected to a lower extent, in part due to lower malaria incidence rates and a better quality of teaching [34]. There has been a recent increase in peri-urban malaria in Latin America, caused by unplanned urbanization of developing cities such as Manaus [35], and malaria remains a major problem in rural areas. The immigrants colonizing the Brazilian Amazon, looking for mining, agriculture and timber exploitation opportunities, are similar in terms of socioeconomic status and profile to the local inhabitants. Therefore, the results found in this study probably reflect what happens in other settlements.

### Limitations of the study

The comparison of our data with others is limited by the distinct manner of evaluation of poor performance, since there is no standardized tool which could be applied worldwide due to cultural issues [11]. However, the auithors believe that the study has a high internal validity, because the local evaluation system itself was analysed, reflecting what happens in the real world. Other potential causes of anaemia were not evaluated, such as iron deficiency and intestinal parasitism, which could have some influence on the study outcome [16]. These data refer only to cognitive impairment in school-aged children and provide no information about the impairment in children under five years of age and/or adults with malaria [36].

The previous episodes of malaria before the study were not analysed due to memory bias, and other factors associated with poor performance were not explored.

## Conclusion

Non-severe malaria causes a negative impact on school performance during an academic year in an unstable transmission endemic area in the Brazilian Amazon, with *P. vivax *as the predominating species. Hence, social, economical and cultural development is potentially impaired in these tropical localities.

## Competing interests

The authors declare that they have no competing interests.

## Authors' contributions

SVS designed the project, led field work, interpreted the results and drafted the manuscript. RCRL performed the statistical analyses, interpreted the results and drafted the manuscript. TRAP contributed with data collection in the field. MVGL designed the project, interpreted the results and critically read the manuscript. All authors read and approved the manuscript.
